# Patients’ perception of colorectal cancer surveillance in the community: an exploratory study

**DOI:** 10.1186/s12889-022-14485-y

**Published:** 2022-11-18

**Authors:** Gretel Jianlin Wong, Jerrald Lau, Emily Chew, Wen-Min Chow, Julia Choo, Ker-Kan Tan

**Affiliations:** 1 Saw Swee Hock School of Public Health, National University of Singapore, 12 Science Drive 2 #10-01, Singapore, 117549 Singapore; 2 Department of Surgery, Yong Loo Lin School of Medicine, National University of Singapore, Singapore, Singapore

**Keywords:** Colorectal cancer, Cancer surveillance, Community care, Post-operative, Prevention

## Abstract

**Background:**

All patients who underwent curative resection for colorectal cancer (CRC) are frequently reviewed in tertiary institutions to ensure timely detection of any disease recurrence. There has been no local study that evaluated the feasibility of monitoring their condition in the community as a possible new model of care. This study henceforth seeks to understand CRC patients’ views and receptiveness of having their surveillance consultations conducted in a community setting.

**Methods:**

We convenience sampled Stage I and II CRC patients who were within five years post-operation in the outpatient clinics. An open-ended questionnaire aimed at elucidating their perception towards cancer surveillance in a community setting was administered. Content analysis was used to group and quantify responses from participants.

**Results:**

Twenty-five participants agreed to participate in the study. Only 48% of the participants felt that having phlebotomy procedures in a community or home setting was acceptable. Participants were less willing to be reviewed by a physician who is not their primary surgeon, with only 32% agreeable to seeing a different doctor for surveillance if given a choice. However, most participants were open to having a telephone consultation in place of a physical face-to-face consultation before (72%) and after (76%) going through medical imaging.

**Conclusions:**

Participants remained keen to be managed by their primary surgeons and were hesitant towards having their follow-up surveillance consultations in community and primary care settings. Further studies should be conducted to understand whether these perceptions are generalisable, and if more can be done to change public perception towards the role of community and primary care institutions.

## Introduction

Colorectal cancer (CRC) is the third most common cancer in the world [1] and the commonest cancer in Singapore [2]. Post-operative surveillance follow-up is the standard care for most CRC patients after a curative resection to ensure timely detection of disease recurrence.

Stage I and II (early-stage) CRC patients are recommended to get reviewed every three to six months until five years post-operation [3]. With an increasing number of CRC patients being managed, post-operative reviews will result in a higher burden on specialist clinics, but the rate of recurrence for early-stage CRC is less than 10% [4, 5]. Our recent publication showed that majority of CRC recurrences were diagnosed using objective clinical tests (97.5%) [6], raising the question of the need for a physical consultation. As Singapore moves towards a more holistic healthcare landscape, the Ministry of Health has been looking to shift more tertiary clinical load into community and primary healthcare settings to optimise healthcare utilisation and increase cost-economical savings.

There is little empirical data in Singapore on the acceptance of cancer surveillance in community settings. If found to be acceptable, cancer surveillance in community settings can be protocolised as part of the patient care continuum. The purpose of this exploratory study was therefore to assess early-stage, low-risk CRC patients’ perceptions on cancer surveillance in community and primary care settings, juxtaposed with usual care arrangements in tertiary healthcare institutions. We also examined the relationship between doctor and patient, and how this relationship potentially affects the patient’s acceptance of care in a community setting.

## Methods

### Participants

Patients were cluster sampled between September and December 2018 during their post-operative surveillance consultation at the National University Hospital (NUH) Colorectal Clinic. The timeframe was selected as this pilot study was designed alongside the clinic’s operation interest in exploring the feasibility of decanting patients into primary and community care settings. Patients were screened and approached based on the eligibility criteria that they had been (1) diagnosed with early-stage CRC and (2) within five years post-operation for CRC within the sampling timeframe. A convenience target sample of 25 (of the 28 eligible patients) agreed to participate in our study (89.3%).

### Measures

We utilised a self-constructed open-ended questionnaire and recorded verbal responses. The questionnaire attempted to elicit feedback that the participants had towards the current system of follow-up surveillance care in NUH Colorectal Clinic. This was followed by an introduction to the hypothetical scenario of moving post-operative care from specialist clinics to the community and primary care institutions. This hypothetical scenario was presented verbally using details that attempted to make it more relatable to each participant, for example, by using the name of the doctor in charge of their care to ground the scenario. The study measures attempted to ask if early-stage, low-risk CRC patients were:

1. Willing to accept care and peripheral tests in a community setting?
2. Willing to undergo medical investigations without a face-to-face review by a medical doctor?
3. Receptive to having a review with a specialised healthcare professional (HCP) (i.e., case manager or nurse practitioner)?
4. Open to telemedicine as a follow-up option in place of a face-to-face consultation?

Participants’ responses were recorded ad verbum on the open-ended questionnaire at the point of interview and manually coded and common themes identified via content analysis at the end of data collection.

## Results

Majority of the participants were female (56%, *n* = 14), of Chinese ethnicity (84%, *n* = 21), were retired or unemployed (68%, *n* = 17), had a median age of 70 years (range = 52 – 88 years) and had been previously diagnosed with Stage II CRC (80%, *n* = 20). Demographic information, self-reported by participants as part of the questionnaire, can be found in Table 1.

**Table 1:** Demographic of participants

Demographics		Employed		Unemployed		*p*-value
		(*n* = 8)		(*n* = 17)		
		n	(%)	n	(%)	
**Ethnicity**	Chinese	5	62.5%	16	94.1%	0.049
	Malay	1	12.5%	1	5.9%	
	Indian	2	25.0%	0	0.0%	
**Gender**	Male	3	37.5%	8	47.1%	0.200
	Female	5	62.5%	9	52.9%	
**Income**	< 2 k	2	25.0%	17	100.0%	0.001
	2-4 k	4	50.0%	0	0.0%	
	4-6 k	1	12.5%	0	0.0%	
	6-8 k	1	12.5%	0	0.0%	
**Religion**	Buddhism	1	12.5%	7	41.2%	0.217
	Catholicism	0	0.0%	1	5.9%	
	Christianity	1	12.5%	2	11.8%	
	Hinduism	2	25.0%	0	0.0%	
	Islam	1	12.5%	1	5.9%	
	Taoism	0	0.0%	2	11.8%	
	No Religion	3	37.5%	4	23.5%	
**CRC stage**	Stage I	3	37.5%	2	11.8%	0.063
	Stage II	5	62.5%	15	88.2%	
**Age**	51—60	4	50.0%	0	0.0%	0.008
	61—70	3	37.5%	6	35.3%	
	71—80	1	12.5%	8	47.1%	
	81—90	0	0.0%	3	17.6%	

Most participants reported that they were pleased with the current service provided by the specialist clinic. The most suggested improvement was about lengthy waiting time (20%, *n* = 5). 72% (*n* = 18) of the participants reported being accompanied by a caregiver for their initial post-operative surveillance consultations.

68% (*n* = 17) of the participants were unwilling to be seen by a doctor who is not their primary surgeon. Most working participants (87.5%, *n* = 7; of 8) were not agreeable to being seen by a different doctor for surveillance. 44% (*n* = 11) were comfortable with having a specialised HCP conduct their surveillance follow-up. Participants were split on undergoing basic phlebotomy procedures outside of the specialist clinic, with 48% (*n* = 12) of the participants being agreeable to having their surveillance blood tests done in a home visit or community setting nearer to their home. The main concerns raised by participants for this arrangement were the potential increase in waiting time and cost.

The idea of telemedicine via video conferencing replacing a face-to-face follow-up consultation was introduced. Only 16% (n = 4) of the participants were open to the idea—of which 75% were employed. Participants who rejected the idea reported being unconfident in using tele-consultation because of their limited knowledge of technology. Participants were then asked about having a telephone consultation in place of the physical pre- and post-imaging consultations, the latter in place with the caveat that the imaging test results are negative. All employed participants (*n* = 8) were open to the idea, while 58.8% (*n* = 10) and 65.7% (*n* = 11) of the unemployed participants were agreeable to not having pre- and post-imaging consultation respectively. A descriptive list of responses from the participants can be found in Table 2.

**Table 2:** Responses from participants on current care and acceptability of surveillance in the community

Variables		Employed		Unemployed		*p*-value
		(*n* = 8)		(*n* = 17)		
		n	(%)	n	(%)	
**Accompanied for medical appointments**	All the time	3	37.5%	10	58.8%	0.606
	After surgery only	2	25.0%	3	17.6%	
	Not at all	3	37.5%	4	23.5%	
**Complaints about waiting time in clinic**	Yes	1	12.5%	4	23.5%	0.520
	No	7	87.5%	13	76.5%	
**Having a Ddifferent doctor for consultation**	Agreeable	1	12.5%	7	41.2%	0.126
	Not agreeable	7	87.5%	9	52.9%	
**Having Oother HCPs for consultation**	Agreeable	3	37.5%	8	47.1%	0.546
	Not agreeable	3	37.5%	6	35.3%	
**No consult before medical imaging**	Agreeable	8	100.0%	10	58.8%	0.095
	Not agreeable	0	0.0%	4	23.5%	
**No consult after medical imaging**	Agreeable	8	100.0%	11	64.7%	0.159
	Not agreeable	0	0.0%	3	17.6%	
**Phlebotomy at home**	Agreeable	4	50.0%	8	47.1%	0.653
	Not agreeable	3	37.5%	9	52.9%	
**Phlebotomy in the community**	Agreeable	4	50.0%	8	47.1%	0.746
	Not agreeable	4	50.0%	6	35.3%	
**Telemedicine in place of physical appointment**	Agreeable	3	37.5%	1	5.9%	0.053
	Not agreeable	5	62.5%	15	88.2%	

## Discussion

This study demonstrated that challenges remain to be overcome for patients to accept having part of their cancer surveillance performed in the community. Participants were comfortable with the current surveillance care arrangements and were not keen to see a different doctor. As current literature suggests, consistency and standard of follow-up care are valued by cancer patients [7, 8]. Having built rapport with their surgeons through their cancer journey, patients would feel comfortable entrusting their health to these specialists [7]. It was interesting to note that employed participants were more willing to wait for their consultation despite having to take time off work. More can be done to uncover issues and barriers faced by working patients so that the economic cost of taking time off can be minimised.

Another area for investigation is the agreeability of participants to having a specialised HCP see them for surveillance consultations. This may be due to patients’ exposure to the teamlet care model in Singapore’s subsidised primary care institutions (polyclinics). Teamlets consist of physicians, nurses, and care coordinators, and are in charge of care for a fixed group of patients with chronic diseases [9]. The patient may be attended to by any of the HCPs in the teamlet, and patients are aware that their care plan would have been discussed amongst the team before they are attended to, ensuring care continuity. Participants may thus be comfortable with seeing a specialised HCP because of the familiarity with this system.

Participants were split on having surveillance phlebotomy procedures outside of the specialist clinic. One reason may be that cancer patients enjoy heavily subsidised care in specialist clinics. Some participants were therefore concerned that phlebotomy costs would increase drastically should it take place in polyclinics or their homes. Participants were also concerned about longer waiting times as they viewed these services as less efficient. Some participants preferred the one-stop nature of their visit to the specialist clinic, where the phlebotomy procedure was followed by the doctor’s consultation. While home phlebotomy theoretically allows a patient to wait in the comfort of their homes, this may come with an uncertain timeframe.

Another aspect that may have been overlooked is the social aspect these healthcare visits pose, especially for unemployed patients. They would potentially treat attending the specialist clinic like a social routine, much like visiting old friends [10].

Most participants did not like the idea of replacing face-to-face consults with telemedicine via video conferencing. Some unemployed participants were unwilling to learn skills for telemedicine consultations, possibly due to high perceived effort achieving digital literacy requires [11]. Whether the level of rapport will be maintained in a telemedical consult remains undetermined. That said, working participants were more inclined to agree to using telemedicine. Given their work commitments, telemedical consultation provides a good alternative to seeing a doctor without taking time off from work.

The results of this exploratory study highlight difference in perspectives between healthcare providers, policy makers and patients. Whilst shifting tertiary clinical load into the community and primary care settings makes economical and logistical sense, it may not be as well accepted by patients [12]. A clearer understanding of care integration in community surveillance care, emphasising the input of both specialists and primary care professionals, could help patients better accept community surveillance [13]. An in-depth evaluation is necessary to identify barriers and issues faced by patients, such as rapport, adoption of technology, healthcare costs, and reputation of primary care institutions. Despite our limited sample size, this study allowed us to explore the views of patients with the least intensive post-operative surveillance follow-up schedules, giving a snapshot of the concerns of the patients that the healthcare system would need to address in routing cancer surveillance consultations into community and primary care facilities. Addressing these concerns may help increase patients’ acceptance of community surveillance and in turn, the feasibility of moving forward with a care continuum where patients who are stable can be monitored in the primary care and community rather than at the tertiary clinic.

## Conclusion

Early-stage CRC patients are not keen to transfer their care from a tertiary to a primary healthcare setting, despite the low risk of cancer recurrence and standardised surveillance protocols. A combination of assurance from their surgeons and change in perception towards community and primary care institutions may help patients be more accepting of having their surveillance consultations there. Future studies should employ a more robust cohort to uncover the concerns of patients on their surveillance journey and explore how to encourage public perception of primary care institutions as healthcare providers of cancer surveillance care.

## Data Availability

The datasets used and/or analysed during the current study available from the corresponding author on reasonable request.
